# Proteomic Discovery and Validation of Novel Fluid Biomarkers for Improved Patient Selection and Prediction of Clinical Outcomes in Alzheimer’s Disease Patient Cohorts

**DOI:** 10.3390/proteomes10030026

**Published:** 2022-08-01

**Authors:** Shivangi Awasthi, Daniel S. Spellman, Nathan G. Hatcher

**Affiliations:** Merck & Co., Inc., Kenilworth, NJ 07033, USA; shivangi.awasthi@merck.com (S.A.); daniel_spellman@merck.com (D.S.S.)

**Keywords:** neuroproteomics, Alzheimer’s disease biomarker, neurodegeneration

## Abstract

Alzheimer’s disease (AD) is an irreversible neurodegenerative disease characterized by progressive cognitive decline. The two cardinal neuropathological hallmarks of AD include the buildup of cerebral β amyloid (Aβ) plaques and neurofibrillary tangles of hyperphosphorylated tau. The current disease-modifying treatments are still not effective enough to lower the rate of cognitive decline. There is an urgent need to identify early detection and disease progression biomarkers that can facilitate AD drug development. The current established readouts based on the expression levels of amyloid beta, tau, and phospho-tau have shown many discrepancies in patient samples when linked to disease progression. There is an urgent need to identify diagnostic and disease progression biomarkers from blood, cerebrospinal fluid (CSF), or other biofluids that can facilitate the early detection of the disease and provide pharmacodynamic readouts for new drugs being tested in clinical trials. Advances in proteomic approaches using state-of-the-art mass spectrometry are now being increasingly applied to study AD disease mechanisms and identify drug targets and novel disease biomarkers. In this report, we describe the application of quantitative proteomic approaches for understanding AD pathophysiology, summarize the current knowledge gained from proteomic investigations of AD, and discuss the development and validation of new predictive and diagnostic disease biomarkers.

## 1. Introduction

The absence of therapeutics to halt or even slow the progression of neurodegenerative diseases such as Alzheimer’s disease (AD), Parkinson’s disease (PD) and frontotemporal lobar degeneration (FTLD) is a vast unmet medical need that continues to worsen as the population ages. A major challenge facing drug development for neurodegenerative diseases lies in the early detection of disease to select patient groups whose pathology have not yet progressed beyond the potential for intervention and to enable the early prediction of drug efficacy in clinical trials that test novel therapies [1,2]. There has been significant investment in the development of neurodegenerative disease biomarkers—with innovations in AD research as an example—that include fluid biomarkers measured in cerebral spinal fluid (CSF) and the discovery of PET ligands that enable the determination of both amyloid beta and tau protein pathologies. While clinical biomarkers have yet to be discovered for other major neurodegenerative diseases, such as PD and FTLD, even in the example of AD, where tau and beta amyloid clinical biomarkers of disease state have been validated, the utility in predicting clinical outcomes is yet to be demonstrated. Thus, there remains a need for the additional discovery of biomarkers for the early detection of the neurodegenerative disease pathology, progression rate, prediction of conversion to late-stage pathology, and assessment of clinical efficacy in drug trials. Changes in the protein content of patient fluids, such as CSF, can provide insights into the neurodegenerative disease state and offer a rich potential source for the discovery and validation of putative biomarkers in a readily accessible fluid specimen. Proteomic analyses of patient CSF can provide an unbiased discovery strategy to identify disease-related changes in the protein content. Modern discovery proteomics approaches follow a variety of analyte identification and quantification strategies to differentiate proteins that are statistically up- or downregulated in patient fluid specimens. These analyses typically utilize extensive analyte prefractionation and separation using liquid chromatography coupled to high-mass-accuracy mass spectrometers, and these approaches follow a variety of analyte identification and quantification strategies from sample preparation to the bioinformatic and statistical modeling of data to differentiate putative protein fluid biomarkers for follow-up validation studies. In this review, we discuss fluid biomarker development from discovery to validation with examples taken from the published studies within AD research. We review the current quantitation workflows and bioinformatic tools for differential biomarker discovery, discuss experimental design considerations for effective comparisons using these unbiased approaches, and close with an overview of the follow-on targeted quantification of biomarker candidates required for final validation using the Alzheimer’s Disease Neuroimaging Initiative (ADNI) Longitudinal Proteomic Changes in CSF studies as an example.

## 2. Clinically Validated Fluid Biomarkers for AD

### 2.1. AD Epidemiology

Alzheimer’s disease (AD) is recognized as a global public health priority by the World Health Organization. Current estimates in the United States show that a total 6 million Americans are living with AD, with future projections of incidences expected to rise to nearly 13 million by 2050 as advanced age is the primary contributing factor for AD and other dementias, and occurrence is predicted to increase with increasing global life expectancies.

AD is the most common type of dementia, accounting for 50–70% of all cases. AD is the sixth leading cause of death in the country, and while deaths from heart diseases have decreased by 7.3% between 2000 and 2019, AD mortalities have increased by 145%. In 2021, healthcare estimates for AD and other dementias are $355 billion, and by 2050, these costs could rise as high as $1.1 trillion [3]. Beyond costs, AD patients suffer from long-term, progressive losses in memory, cognitive function, speech, reasoning, personality, and motor functions that can result in significant ancillary burden for relatives and health care providers.

### 2.2. AD Pathology and Diagnosis

A neuropathological feature shared by most common neurodegenerative diseases includes the aggregation or deposition of certain proteins in neurons or extracellular spaces, which is often termed as “proteopathies.” The primary hallmarks of AD pathology are amyloid beta Aβ-containing plaques and tau-containing neurofibrillary tangles [4]. Although the roles of Aβ and tau pathology in neurodegeneration have not been elucidated, the deposition of extracellular plaques containing Aβ peptides have been linked to synaptic and neuronal defects, a phenomenon termed as “amyloid cascade hypothesis” [5]. The transmembrane amyloid precursor protein (APP), upon cleavage by β-secretase (BACE1) and γ-secretase, releases insoluble Aβ42 peptides that form aggregates that accumulate into larger fibrillar structures, which form the defining plaque tissue pathology within the brain [6]. Tau protein acts to stabilize microtubules that are important for intracellular transport in axonal and dendritic processes and maintains neuronal cytoskeleton integrity. Aberrant phosphorylation and truncations of tau proteins form the major components of neurofibrillary tangles [7,8]. Hyperphosphorylated tau inhibits assembly and disrupts microtubules and impairs the axoplasmic flow, as well as causing a loss of neuronal connection [9]. It has also been shown that hyperphosphorylated tau can spread in a prion-like fashion and be taken up by surrounding neurons via endocytosis [10].

The criteria for AD diagnosis were first published by the National Institute on Neurological and Communicative Disorder and Stroke and the Alzheimer’s Disease and Related Disorders Association (NINCDS-ADRDA) that helped the clinicians differentiate between symptoms as “unlikely”, “probable”, “possible”, or “definite” AD [11]. These clinical exclusion criteria required a neuropathological examination upon death to ascertain a “definite AD” diagnosis as no imaging or fluid biomarkers were yet available. In 2007, the international working group (IWG) published the first research criteria for the diagnosis of prodromal AD, which allowed for the use of biomarkers like CSF Aβ42, tau, volumetric MRI, and amyloid PET [12]. The National Institute on Aging (NIA) and the Alzheimer’s Association (NIA-AA) published guidelines updating the 1984 clinical diagnostic criteria [11]. These guidelines elaborated the AD dementia beyond memory loss by classifying developmental disease stages beginning with an early and long-lasting asymptomatic pre-clinical stage and followed by mild cognitive impairment (MCI) before progressing to a final stage defined as AD dementia. Based on the amyloid cascade hypothesis, AD pathology can begin to set in several years before the onset of any clinical symptoms. MCI was introduced as a term to denote the transitional zone between cognitive decline seen in normal aging and cognitive dysfunction seen in AD [13]. Updates have been published for MCI due to AD [14], as well as AD dementia [2,15]. In recent years, there has been a focus on describing a phase that precedes MCI, i.e., the pre-MCI phase [16,17], although no standard definition is available and efforts have been directed to analyze the symptomatic debut of AD. AD patients with early onset (age < 65 years, EOAD) and late onset (age > 65 years, LOAD) present different cognitive and brain atrophy profiles. EOAD patients present multimodal cognitive dysfunction, performing poorly in visuo-spatial, speech, logical, and attention tasks while memory impairment is not dominant. LOAD patients, on the other hand, exhibit episodic memory impairment as a central symptom even in the early MCI phase [18]. Longitudinal studies have demonstrated a more rapid decline in EOAD than LOAD [19].

The 2011 NIA-AA updates were made followed by considerable advancements in knowledge regarding disease pathology as well as the maturation of clinical, imaging, and research techniques. These new guidelines added the use of CSF and imaging biomarkers as secondary diagnostic techniques, although these were limited primarily to supportive tools in clinical research. The IWG criteria was updated in 2014, where the CSF biomarkers, low Aβ42 combined with high total tau or phospho-tau, had more of a primary role while volumetric MRI and PET were to be used as secondary tools to monitor neurodegeneration [20]. In 2018, NIA-AA defined a new “research framework”, A/T/N, which identified imaging and CSF biomarkers as valid diagnostic tools [21,22]. This revision of relying on biomarkers as indicators of disease progression reduces the dependence on patient biopsies or autopsies to obtain a definite diagnosis. Under this A/T/N framework, AD diagnosis is assessed by the presentation of amyloid pathology (A+, by amyloid PET or in CSF), by the presence or absence of tau fibrillation (T, by PET or phospho-tau in CSF), and the extent of neurodegeneration (N, by structural MRI or total tau in CSF). The A/T/N framework allows for the addition of new biomarkers as and when they become available to better support the overall characterization of AD. This new protocol distinguished the three CSF core biomarkers of AD as per the pathological mechanisms to which they contribute.

### 2.3. Clinical Utility of CSF Aβ42, Total Tau, and Phospho-Tau

AD patients exhibit a lower CSF Aβ42 concentration when compared to controls [23,24,25]. A significant increase in CSF total tau and phospho-tau (p-threonine-181) in AD dementia patients has also been shown and replicated by many studies [23,26]. Since plaques and tangles are at the core of disease biology, Aβ42, total tau, and phospho-tau (p-tau) are referred to as the core biomarkers. Compared to healthy controls, AD patients exhibit low CSF levels of Aβ42, whereas total Aβ is unchanged. These values are often expressed as an Aβ42/Aβ40 ratio that accounts for intra-individual variations. Additionally, AD patients exhibit high CSF levels of total tau and phospho-tau. This distribution of Aβ and tau in CSF is common among individuals with AD and is generally referred to as the “AD signature” [27].

CSF Aβ42/Aβ40 as well as total and p-tau signatures have shown promise for the early detection and prognosis for risk of progression. In a three-year longitudinal study following early MCI and healthy age-matched controls, Hansson and co-workers monitored CSF levels of tau and Aβ1–42 and were able to demonstrate a diagnostic sensitivity of 95% and a specificity of 83% at discriminating AD in patients with MCI as well as the increased risk of progression to AD in MCI individuals exhibiting abnormal total tau and Aβ1–42 at baseline measures [28].

Recent data suggest a critical role of hyperphosphorylation of tau occurring at different stages of disease progression that follows distinct trajectories over time. A comprehensive investigation that measured phosphorylation occupancy at multiple tau residues in CSF showed that tau phosphorylation is dynamic and begins once the aggregated Aβ pathology is seen, changes over two decades of tracking the clinical progression of the disease, and goes down significantly in a site-dependent manner near the onset of cognitive decline [29]. Specifically, CSF p-threonine-217 levels are closely related to amyloidosis at asymptomatic and symptomatic stages and has been proposed as a biomarker to monitor AD pathology [30]. A follow-up study found a positive correlation between p-threonine-217 levels in plasma and CSF in clinical samples [31]. Another longitudinal analysis of plasma p-threonine-217 levels showed clinically relevant changes that correlated with the pre-clinical disease stage, MCI as well as MCI to Alzheimer’s disease converters [32]. A recent study also highlighted the localization of p-threonine-217 in neurofibrillary tangles (NFTs), neuropil threads, and multi-vesicular bodies in the post-mortem AD brain tissue, thereby strengthening the potential of p-threonine-217 to be used as a biomarker [33].

### 2.4. The Unmet Medical Need

A true disease biomarker should highlight the activity of the disease process, predict outcomes, and provide a pharmacological readout to therapeutic intervention. For a biomarker to be translated to a validated clinical test, it should be easy to use, cost-efficient, reproducible, and should provide high sensitivity and specificity [34]. These points were emphasized further by both the Food and Drug Administration (FDA) and European Medicines Agency’s (EMA) Committee for Medicinal Products for Human Use (CHMP) in the context of AD biomarker discovery [35,36].

Currently, AD biomarkers can be divided into several categories: those that are derived from neurogenetics [37,38], neuroimaging, and neurophysiology [39,40], and biochemical fluid biomarkers (CSF [41] and blood-based [42]). However, the success of such a multimodal approach is yet to be determined and needs additional development in terms of specificity and selectivity [43].

The accuracy of AD diagnosis in the clinic remains low and experienced neurologists and clinicians face challenges diagnosing AD in subjects with dementia. The rates of accuracy of AD diagnosis have been reported to be generally low, with sensitivities ranging between 71% to 88% and specificities from 44% to 71% [44]. It was also reported that the rate of misdiagnosis of AD can be as high as 20% [45]. This may be due to the presence of other co-morbidities, such as diabetes and hypertension that may impact Aβ levels [46]. The 2015 ADNI study showed that over 50% of the AD diagnosed patients show other pathologies as well [47].

The effects of AD span beyond the neurological system as it is known that AD patients show physical decline as well. These systemic manifestations are in part driven by gradual and progressive cognitive and behavioral failures linked to neurodegeneration. The high degree of heterogeneity observed in the biological genotypes as well as behavioral phenotypes is evident by a great variety of pathological lesions, kinds of clinical symptoms, onset age, and types of behavioral manifestations. This well-documented information along with the presence of a plethora of molecular mechanisms involved in AD pathophysiology emphasizes the multifactorial nature of the disorder. Although, the clinical accuracy of CSF Aβ42, tau, and phospho-tau CSF levels for diagnosis of the “AD signature” is robust, there are issues pertaining to sensitivity and specificity. There are examples of individuals who lack the AD CSF signature but have developed dementia. Additionally, it has been reported that tau CSF levels can increase in normal aging and can fail to discriminate AD from other forms of dementia like vascular dementia (VaD), frontotemporal dementia (FTD), and Creutzfeldt–Jakob disease (CJD) [48,49]. Moreover, there is clinical evidence that Aβ dysregulation may contribute to cell dysfunction and pathologies other than AD [46].

Altered levels of Aβ42, tau, and phosphorylated tau in CSF have been accepted to varying degrees as a diagnostic tool for AD in different countries. Even though IWG and NIA-AA have advocated for the use of these fluid biomarkers to add diagnostic value, they do not endorse the use of these biomarkers for routine diagnosis as there is a need for further research, qualification, and standardization of such tests for use as diagnostics [12,15]. CSF levels of Aβ and tau are currently considered supportive indicators within a broader clinical phenotype [2].

Alterations in CSF Aβ and tau CSF have utility in discriminating early MCI and offer potential in relating disease progression, but these changes occur over a relatively short increment of disease and thus have inherent limitations for early detection and prognosis, as well as for use as surrogate efficacy biomarkers in drug trials. For example, CSF Aβ42, tau, and phospho-tau show minimal changes when patients progress from MCI to AD dementia [30,31] or when patients are in the clinical phase of AD dementia [32]. This indicates that the alteration of normal to pathological levels of these CSF biomarkers precedes clinical symptoms and occurs during the pre-clinical asymptomatic phase of the disease. Since these biomarkers are stable over MCI and AD dementia, they don’t always allow for AD prognosis and accurate disease staging. Moreover, clinical trials currently rely on relatively late-stage indicators of disease where levels of CSF Aβ and tau may be maximally altered and no longer sensitive to therapeutic intervention.

Given these limitations, there is an urgent need to discover additional CSF biomarkers that cover a greater portion of AD disease progression. To support advances in AD intervention, prevention, and treatment, the discovery of new diagnostic biomarkers is needed at the earliest stage in the disease continuum where the intervention is expected to provide the most long-term benefits. Additionally, new fluid biomarkers would benefit the selection of prodromal patients with a greater risk for developing AD and therefore could aid in appropriate patient selection for clinical trials. Within clinical trials, there is a need to determine target engagement and potential disease modification of therapeutic candidates. Thus, additional biomarkers likely can be used as surrogates of clinical efficacy across the specific timepoints or stages of disease within selected patient populations.

## 3. Mass Spectrometry-Based Discovery Proteomics

Recent advances in proteomic approaches using state-of-the-art mass spectrometry are now being increasingly applied to elucidate disease mechanisms, identify drug targets, and to identify novel disease biomarkers [50]. Neuroproteomics has emerged as a subcategory of proteomics and has been utilized extensively in the study of neurodegenerative diseases to identify potential biomarker candidates with diagnostic, prognostic, and therapy predictive utilities as demonstrated by the growth rate of publications in this area [51]. The Human Proteomics Brain Proteome Project (HUPO BPP, http://www.hbpp.org/, accessed on 17 June 2022), an international initiative under the global Human Proteome Organization (HUPO), aims to further consolidate proteomic efforts to facilitate scientific discussions and collaborations and translate such efforts to clinical testing in various neurodegenerative disorders, including AD. The following sections will review proteomic applications using quantitative mass spectrometry-based approaches and the current knowledge gained from proteomic investigations of AD with a focus on the discovery and validation of CSF biomarkers related to AD progression.

Proteins control the synthetic, catalytic, and regulatory biochemical processes and often assume a higher-structured order referred to as a proteome [52,53]. The large-scale systematic measurement of proteomes to generate biological insights about a system is referred to as proteomics. Mass spectrometry (MS)-based approaches have emerged for reliable and exhaustive investigation into the composition and function of the proteome as an integrated system [54,55,56,57]. Liquid chromatography coupled to tandem MS (LC MS/MS)-based proteomics analysis may be comprised of simply identifying the proteins, looking into the nature and location of the post-translational modifications (phosphorylations, glycosylations, etc.) [58,59], measuring the dynamic quantitative changes between conditions, or studying the protein conformations or interactions in the context of biological signaling pathways [60,61].

Mass spectrometry has been successfully applied to proteomics analysis due to its inherent specificity of identification and its exceptional sensitivity. The field of mass spectrometry has seen tremendous growth in the past four decades with regards to the kinds and configurations of ionization sources, mass analyzers [62,63], and ion detectors, which has significantly impacted the proteome analysis [64]. The most significant of these developments include the implementation of “soft” ionization techniques electrospray ionization (ESI) [65,66] and matrix-assisted laser desorption/ionization (MALDI) [67]—although they were developed in the 1980s, they remain prevalent for biomolecular analysis today. These “soft” ionization techniques are well suited for proteins and peptide analysis as these are polar, non-volatile, and unstable analytes that need to be ionized without extensive degradation. MS-based proteomics has led to the successful identification and accurate quantification of many proteins in complex biological matrices, thereby advancing the understanding of the cellular signaling pathways, characterizing the dynamics of protein–protein interactions in varied cellular states and locations, shedding light on the complex disease mechanisms, and providing unique biological insights [56,64,68].

### 3.1. Bottom-Up Shotgun Proteomics (DDA vs. DIA)

In a typical bottom-up proteomics experiment, sequence specific protease (trypsin is most common) cleaves proteins into peptides, which are separated through a reverse-phase liquid chromatographic (RP LC) system (at nanoliter/minute flow rates [69]), and are thereafter subjected to electrospray ionization (or nanospray (NSI) [70]) followed by a full MS1 scan [54,70]. The top-most abundant ions are selected from the MS1 scan, fragmented by collision with gases like nitrogen or argon (most commonly by collision-induced dissociation (CID) or higher energy collisional dissociation [71]), followed by the acquisition of a MS2 scan of the resulting fragments (referred to as data-dependent acquisition (DDA) or “topN”). A single proteomics experiment will often have thousands of peptides being eluted off the LC column while the mass spectrometer generates the tandem spectra for the same. This tandem mass spectra provides the necessary information for peptide sequencing [72].

Standard instruments for DDA proteomics experiments include high-resolution and high-mass-accuracy analyzers [73,74,75]. Mass analyzers such as the Orbitrap and the QTOF provide detailed structural information for accurate peptide and protein identification at acquisition speeds that can enable the identification of hundreds of proteins in a single LC run, but comprehensive coverage of the proteome is far from complete in these analyses. The dynamic range of protein expression in a cellular system can range from seven to eight orders of magnitude [76,77,78]. One study found that in a typical shotgun LC MS/MS run, approximately 100,000 “peptide-like” features elute, but the current instrumentation is limited in its sequencing speed, sensitivity, and precursor ion isolation capabilities [79]. Various fractionation strategies at the protein or peptide level are often applied to overcome instrument limitations and delve deeper into the proteome (see Section 3.4).

Whereas DDA selects the most abundant peaks in MS1 for fragmentation, Data Independent Acquisition (DIA) workflows aim to acquire complete MS/MS sequencing data systematically throughout the chromatographic run, independent of the detected analytes in the MS1 scan. The isolation windows for precursor ions in MS1 are set to cover the entire m/z range of the eluting peptides and can therefore generate extensive MS/MS maps for all the observable analytes in a sample. Opening of the precursor isolation windows leads to chimeric MS2 spectra originating from co-fragmentation of multiple peptides. As a large-scale discovery method, DIA can potentially overcome the under-sampling restrictions of DDA and limitations with the number of analytes that can be analyzed by selected/multiple reaction monitoring (SRM/MRM), without compromising the analytical and quantitative performance [80]. However, proper interpretation of DIA data can be cumbersome because of the complex MS2 data that contain data from multiple peptides and can therefore be difficult to analyze.

### 3.2. Quantitative Proteomics for Differential Feature Identification

A major advantage of mass spectrometry-based global proteomics is its capability to quantify proteins in a biological system. These quantitative measurements of protein abundances help build a functional network and provide a snapshot of protein changes associated with different states. These altered levels of proteins may provide clues for potential drug targets or disease biomarkers. MS-based quantitative proteomics can be broadly divided into two groups: (1) Relative quantitative proteomics, where protein abundances are compared between two or more samples/conditions. The advancements of mass spectrometry instrumentation and bioinformatics today allow for the quantification of protein expression changes on a few to tens of thousands of proteins under normal or perturbed conditions. (2) Absolute quantitative proteomics deals with measuring the absolute abundances of proteins in samples. A variety of approaches that are utilized to carry out quantitative proteomic experiments are discussed in the following sections.

#### 3.2.1. Labeling Strategies for Quantitative Proteomic Comparisons

Most biological studies involve the measurement of protein quantities in a sample beyond generating long lists of protein identifications. DDA proteomics also provides the opportunity to extensively quantify the identified proteins relative to other samples. Though it is important to note that bottom-up studies suffer from technical limitations, i.e., equal amounts of multiple peptides will not generate an equal MS signal, due to differences in ionization efficiencies that lead to ion suppression. To overcome this challenge, various quantitative proteomic strategies have been developed that are based on chemical isotopic labeling [81,82], metabolic isotopic labeling, and isobaric tagging [83]. The underlying quantification strategy takes advantage of the similar physical properties between isotopomers, which result in minimal differences in ionization and near-identical ion suppression effects. Therefore, the labeling would provide good accuracy for quantification. The labelled spike-in proteins/peptides have an important application in measuring the absolute abundances of endogenous proteins in biological samples, assuming the labelled standard is present in the same amount in all the samples [84].

Stable isotope labeling by amino acids in cell culture (SILAC) is a popular MS1-based approach that is extensively applied to cell culture-based relative quantification experiments, which provides the highest accuracy and precision in a shotgun proteomics study. MS2-based relative quantitation approaches like isobaric tags have been commercialized as relative and absolute quantitation (iTRAQ) or tandem mass tag (TMT) reagents. These tags provide multiplexing capability and can analyze up to 16 samples at once. Although, the high throughput comes at the cost of ratio distortion, which may lead to co-isolated precursors contaminating the tandem spectra. Technological advancements as well as mature data analysis platforms help curb this problem [85].

#### 3.2.2. Label-Free Feature Extraction and Quantitation

Despite label-based approaches being the gold standard for MS-based quantitative proteomics methods [86], label-free quantitation (directly measuring the MS1 or MS2 responses) has gained momentum in the last decade. In the label-free quantitation workflow, no label is introduced in the sample, all the samples are run in separate LC MS experiments, and independent peptide parameters are then used to carry out the analysis of the relative protein abundances. Label-free quantification can be carried out either by using algorithms based on extracted ion chromatograms (XIC), e.g., using peak areas computed from MS1 intensity measures or spectral counting algorithms based on MS2 spectra [87]. Label-free methods are easy to implement, require no additional sample processing steps, and are not limited by the number of samples. Although this approach does require complicated normalization and chromatographic alignment to account for retention time shifts and the false matching of peptides across samples, it has become more popular and versatile due to its flexibility, developments in high-resolution and fast-scanning orbitraps [73], and advancements in software solutions for the alignment of multiple MS runs [88].

### 3.3. Data Processing and Bioinformatics

As mass spectrometric instruments have advanced over the years, the data generated from discovery proteomic experiments has grown increasingly intricate. The bioinformatics analysis of this raw MS data requires sophisticated algorithms that can confidently assign peptide identifications, through an untargeted spectrum-centric search, to protein identifications [89,90]. The bioinformatic analysis of DDA proteomic data involves the transformation of the MS raw data into suitable formats to be used as an input for the search algorithms; the experimental spectra are then assigned identifications by matching to a protein sequence database or via a spectral library search or by de-novo sequencing. Different database search engines employ unique spectrum algorithms for peak picking, peptide sequencing, and scoring MS2 spectra. The final step involves assigning peptide and protein identifications by applying statistical criteria. The threshold of protein detection is then determined by the calculation of a false discovery rate (FDR). The most common method of calculating FDR includes the construction of a decoy database that essentially consists of the same protein sequences as the regular database, but these entries have been reversed or scrambled. The FDR is usually fixed at 1% or 5% for typical DDA database searches. Confident protein identification depends on instrument mass accuracy, statistical methods applied for data normalization, missing value imputation, and probabilistic scoring used for peptide-to-protein correlation [56,59,60]. This process of matching raw spectral data within a database or spectral library is defined as a “spectrum-centric search” methodology [80].

For DIA peak assignments, a targeted chromatogram extraction “peptide-centric” data query strategy [91] was implemented. This approach relies on the prior acquisition of MS2 spectral libraries that are then used to uniquely isolate the target peptide through the extraction of the product ion chromatograms and by assessing the peptide co-elution. Theoretically, any target peptide from the newly-acquired data can be searched in this “peptide-centric” approach from the DIA datasets. This approach is intended to address the complicated, chimeric DIA MS2 spectra, as only the chromatographic characteristics like co-elution, peak shape, and intensity match with the assay library are being utilized to assign peptide identity. Therefore, the issue of co-elution and co-fragmentation of several peptides in MS1 becomes irrelevant [80].

### 3.4. Analytical Instrument Considerations

Reversed-phase LC is the most common mode of peptide separations employed in proteome studies. It is critical to carry out the effective LC separation of peptides for a variety of reasons. First, the precursor intensity needs to be above the background noise and high enough for it to be selected for MS2 in DDA analyses. Second, the effective resolution of peptides with similar m/z values is necessary to generate unique and non-chimeric MS2 spectra for peptide identification and to limit potential opportunities for ion suppression. In this regard, one of the most significant enhancements in sensitivity was achieved by coupling nano-electrospray [92] to reverse phase nanoflow LC (nLC). Lowering of the flow rate produces smaller ESI droplets, thereby leading to more effective desolvation, ionization, and mass spectrometric sampling. The online coupling of nLC to nESI-MS may lead to a 100–1000-fold improvement in sensitivity because ESI is a concentration-dependent ionization technique. When the sample amount is a limiting factor, lowering the LC column inner diameter and buffer flow to a nanoliter per minute provides the largest gains in the global proteome coverage and quantitation through improved peak capacity and ionization efficiency [93].

Established chromatographic technological improvements, such as ultrahigh pressure liquid chromatography (UPLC) [94], reduction in resin particle size [95], and the introduction of column frits [96], have also benefited the shotgun proteomic analysis. Monolithic nanoLC columns packed with continuous porous beds provide a rapid and efficient peptide separation without the back-pressure limitations associated with the smaller particle size. Silica-modified monolithic columns have demonstrated superlative performance through the robust identification of over 6000 proteins from HeLa cell lysates [97]. The application of long-gradient (LG) separations coupled to MS has also been proved to be an effective strategy for diving deeper into the proteome. A LG-SRM study using a 150-cm long LC column and a gradient time of 300 min showed a significant reduction in the background interference and an 8–100-fold improvement in the LOQ of the target analytes in serum [98]. A systematic analysis of LG-SRM showed the superior performance of the method by demonstrating lower ng/mL LOQs in abundant protein-depleted plasma [99].

To improve the depth of proteome coverage, a multidimensional separation approach is often utilized to better resolve peptides from complex mixtures and reduce sample complexity [100]. The two-dimensional LC separation before MS (2D-LC MS) provides separation orthogonality, allowing the resolution of components that would have otherwise co-eluted in a single dimension, and thereby improving the overall peptide peak capacity [101]. The first application of 2D-LC MS utilized a strong cation exchange (SCX) in the first dimension. SCX can be carried out using a salt gradient [102], where separation of the peptides is based on their total charge, or using a pH gradient [103], where peptides are resolved according to their isoelectric points (pI). SCX offers ease of use because of its buffer compatibility with MS and can therefore be set up as an online 2D-LC platform [104]. RP-RP has also been used as a 2D-LC approach, where altering the mobile phase pH in the first dimension can improve the orthogonality of separation [105]. Usually, the selectivity of separation is altered by using a high pH in the first dimension followed by a low pH in the second. Hydrophilic interaction chromatography (HILIC) has been used to separate polar and hydrophilic peptides and results in an elution profile opposite to that of RPLC [106]. Zwitterionic HILIC (ZIC-HILIC) was developed and evaluated as a multidimensional separation approach and was found to resemble the 2D SCX separation [107]. Electrostatic repulsion–hydrophilic interaction chromatography (ERLIC), a variation of HILIC, has been shown to perform better than SCX as an orthogonal separation technique [108]. ERLIC has also been applied to explore the phosphoproteome—as well as glycoproteome—from rat kidneys [109].

### 3.5. Emerging Technologies

Alternate post-ionization separation techniques like ion mobility (IM) have recently started gaining prominence in the field of proteomics. Two variations of IM interfaces, namely high-field asymmetric waveform ion mobility spectrometry (FAIMS) and trapped ion mobility spectrometry (TIMS), have been adapted on orbitraps and Q-TOFs, respectively, which aim to provide an additional dimension of separation to facilitate a more comprehensive proteome coverage. FAIMS has been reported to overcome the challenges presented by the co-fragmentation and co-isolation of ions in large-scale proteomics, thereby showing an improved sensitivity for low-abundant species [110]. TIMS coupled with Parallel Accumulation-SErial Fragmentation (PASEF) [111] is the mode of operation for the new timsTOF Pro mass spectrometer and has demonstrated a higher sequencing speed without the loss in sensitivity when applied to a single-shot proteomic experiment [112]. IM offers the ability to overcome co-eluting isobaric precursors as well as the challenges associated with co-isolated and co-fragmented “chimeric” spectra. This can in turn facilitate a more accurate quantification for survey scan-based approaches by eliminating the presence of confounding ions.

Alternate data-acquisition techniques, like BoxCar, have also been recently introduced, and show significant improvement in the ion-beam sampling capacity at the MS1 level by distributing the ions over multiple narrow mass-to-charge segments in the C-trap to increase ion-injection times for lowly abundant species. Using this technique, the authors showed a 90% coverage of cancer cell proteome [113] without requiring extensive offline fractionation.

In the last decade, capillary electrophoresis coupled to mass spectrometry (CE-MS) [114,115] has emerged as a complementary separation technology and has been successfully applied to proteomics for peptide measurements [116], intact protein analysis [117], and biomarker discovery [118]. CE offers many advantages over the traditional RP LC, namely, lower overall run time, lower sample consumption, and better sensitivity. The lower sample consumption has enabled CE to be successfully applied for PTM quantification, e.g., phosphorylation without enrichment [119] and two-plex stable isotope labelled quantification of N-glycans [120].

## 4. Biofluid Sample Preparation

### 4.1. Considerations for Sample Integrity

One of the reasons why the use of CSF biomarkers is challenging in clinical practice is due to the high variability found in the concentrations calculated between different clinical centers. Such lack of reproducibility can be partly attributed to the adoption of different pre-analytical procedures and sample handling steps. To eliminate the confounding effect of the sample processing steps, there is a need to establish standardized operating procedures.

Pre-analytical factors like specimen collection, hemolytic contamination of samples, sample handling, storage conditions and temperature, sample stability over freeze–thaw prior to processing, and batch-to batch variability of the kits utilized are critical methodological issues that need consideration (see reviews [121,122,123,124,125]). The volume of CSF collected can influence the protein concentrations, although the spinal cord gradient effect on Aβ42 has not been demonstrated—though it is suggested to standardize the volume of CSF withdrawal to 12 mL [126].

Use of polypropylene plastic tubes for CSF storage has also been emphasized over polystyrene to avoid adsorption losses of “sticky” proteins like Aβ [127]. As an example, lower values of all the core AD biomarkers were found when stored in polystyrene or glass tubes [128]. One study showed significant differences in levels of CSF Aβ42, tau, and phospho-tau (threonine-181) when polypropylene tubes from different vendors were employed. It was found that the polypropylene tubes introduced polyethylene contamination to the clinical specimens. Moreover, CSF Aβ422 adsorptive loss in tubes differs significantly from phospho-tau due to the varying degrees of protein hydrophobicity and hydrophilicity [129]. These data emphasize the need for the standardization of the type of tube used for CSF biomarker storage and processing, as adsorptive losses can introduce sample handling artifacts into the clinical specimens. The use of 96-well polypropylene plates has shown similar losses of CSF biomarkers. The manufacturer recommendation of using a pre-analysis plate before finally transferring all the samples to the final analysis plate hardly improves the protein losses. In fact, it was shown in two independent studies that using a pre-analysis plate significantly reduced the levels of Aβ42. These variations further contribute to lowering the interlaboratory reproducibility for the CSF diagnostic tests.

Blood contamination of the CSF is a significant issue and can impact the biomarker analysis because the CSF protein amount is only 0.5% when compared to blood [130]. It has been previously demonstrated that blood contamination of CSF can lead to protein degradation [130] and the lowering of Aβ42 levels due to binding of the protein to plasma proteins [131]. Several labs have also evaluated the effect of time delay between CSF collection and storage. Although, studies have reported stable CSF Aβ42, tau, and phospho-tau levels from 72 h [132] to 5–7 days [133,134] after CSF collection, proteomics experiments have reported that the issue is more severe for serum/plasma proteins than CSF [135].

Multicenter studies have been conducted to analyze the impact of other CSF pre-analytical confounding factors in AD biomarker quantitation [122]. As an example, one study tested different spinning conditions, storage volume variations, and the effects of freeze–thaw cycles. It was found that spinning speeds generally do not influence protein levels except for samples where the total CSF protein content is relatively high. As for the CSF storage volume, a reduction of up to half of the tube capacity led to significant lowering of the Aβ42 concentration, while no effect was seen for tau or phospho-tau. Also, it was concluded that tau and phospho-tau can tolerate up to five freeze–thaws, while levels of Aβ42 tend to decrease if freeze–thawed over three times [122].

### 4.2. CSF vs. Blood

Biofluids do practically meet the expectations for an ideal biomarker, as in they are relatively easy to acquire and more economical to implement. CSF surrounds both the brain and spinal cord and continuously receives a stream of molecules secreted from neurons, glia, synapses, and axons—in fact, 20% of all CSF proteins are estimated to originate in the brain [136]. Given its close contact with the brain and constant interaction with the brain interstitial fluid, CSF is the main fluid of choice as a source of protein/peptide biomarker candidates [137]. CSF is collected in clinical settings via lumbar puncture or spinal tap, and overall safety has been repeatedly demonstrated by exhaustive meta-analyses [138,139]. Although the occurrence of post-procedure headaches and other side effects remain substantially low, the procedure suffers from a poor reputation and a general reservation from patients and clinicians. In fact, it was shown in prospective studies with patients exhibiting cognitive symptoms that the incidence of the well-publicized post-lumbar puncture headache is quite low (<2%) and comparable to the headache risk associated with amyloid PET (1.8%) [140,141].

CSF comprises fluid formed by the ultrafiltration of plasma via choroidal capillaries and partly by the secretion of ependymal cells in the brain choroid plexuses. Although CSF composition matches that of the blood plasma, the total protein content is 50–100-fold lower (CSF protein concentrations range from 0.2–0.7 mg/mL when compared to 50–70 mg/mL in plasma/serum [142]). Blood-based protein measures offer a less invasive and cost-effective strategy but present several challenges for proteomic analyses. From a technical perspective, biofluid proteome is highly heterogenous because of the presence of post-translational modifications, alternative splicing, and conformational and structural proteoforms [143]. Second, the dynamic range of protein expression in plasma/serum ranges from albumin at 50 mg/mL to signaling molecules in the range of lower pg/mL (10–12 orders of magnitude), which can further be aggravated by CNS-specific protein abundances being washed out by proteins from peripheral sources [143,144]. Most notably, over 80–90% of the CSF/plasma proteome [145] is dominated by high-abundance proteins (albumin, IgG, transthyretin, transferrin, α1-antitrypsin, apolipoprotein A, alpha-1-acid glycoprotein, haptoglobin, α2-macroglobulin, complement C3 [146]), which severely conceal the more informative low-abundance proteins [147]. The CSF content is dominated by albumin, which makes up 60% of the total protein and is much higher than that in plasma. Moreover, CSF contains other highly abundant proteins, e.g., cystatin C and prostaglandin D2 synthase, that are being synthesized in CNS [148]. Therefore, because of the large differences in protein concentration and their well-known complexity, current proteomic techniques are limited to a small portion of biomarker-appropriate biofluid proteomes [149]. Global initiatives through the Alzheimer’s Association’s International Society to Advance Alzheimer’s Research and Treatment (ISTAART) have been undertaken to standardize both pre-analytical as well as analytical guidelines and tools for AD blood-based biomarkers as part of the Alzheimer’s Association Professional Interest Area (PIA) on Blood-Based Biomarkers (BBB-PIA) [150].

### 4.3. Immunodepletion

To improve the depth of the biofluid proteomic analysis, different strategies for the removal of the most abundant proteins are employed [151]. Affinity depletion of the most abundant proteins and immunoenrichment of the medium and lowly abundant proteins using a combinatorial peptide ligand library are the two most applied approaches. The immunodepletion strategy involves removing the highly abundant proteins by loading the biofluid samples onto a depletion column, where antibodies specific to these proteins capture them, while the lowly abundant proteins are collected in the flow through. Immunodepletion kits have been available in the spin column format for decades now, e.g., Vivapure anti-HAS kit (Sartorius, Göttingen, Germany) and Qproteome (Qiagen, Hilden, Germany). Among the most employed affinity depletion columns [152] are Multiple Affinity Removal Systems (MARS, Agilent, Santa Clara, CA, USA) for depleting the 2, 6, 7, or 14 most abundant proteins; Seppro-IgY-14 (Sigma-Aldrich, St. Louis, MO, USA); and ProteoPrep 20 (Sigma-Aldrich). It has been shown that the LC-based techniques are far superior to the spin columns in terms of replication, amount of carry-over, and non-specific binding [153]. The alternate strategy of relatively enriching the low and medium abundance proteins is based on the concept of a combinatorial peptide ligand library approach, available as ProteoMiner (Bio-Rad, Hercules, CA, USA) [154], which is conceptually complementary to the depletion columns. In this approach, biofluid proteins will bind to immobilized hexapeptide ligands based on the bead capacity. Since the highly abundant proteins will quickly saturate their binding ligands, therefore limiting their binding capacity, the excess copies will be washed off. In comparison, the lowly abundant analytes do not saturate their ligands and will become relatively “concentrated” on the beads. This leads to the reduced dynamic range of the biofluid proteome or “equalizing of the protein concentrations” [155]. Both approaches, affinity depletion and ligand library, have been successfully applied to improve the CSF as well as plasma proteome coverage [151]. A study performed a side-by-side comparison of these two pre-analytical techniques using CSF and found that the MARS-14 column (773 identified CSF proteins) shows a superior qualitative performance over ProteoMiner (611 identified CSF proteins) [156].

## 5. Experimental Design

### 5.1. Analytical Validation, Optimization, and Quality Control

Despite the heavy investment of resources in the area, very few of these candidates pass clinical validation and make it into clinical practice. Common to all ‘omics’ studies, discovery proteomics generates several features (proteins) and are generally labor and time intensive, which leads to the analysis of a limited number of samples. These shortcomings, put together, present challenges both at the step of sample selection as well as the statistical analysis of the resulting data. Therefore, to conduct robust biomarker discovery experiments, special emphasis is needed on defining the study rationale and statistical experimental design.

Much has been written on the importance of experimental design in clinical biomarker discovery through ‘omics’ technologies [157,158]. Special attention needs to be paid to sample selection to avoid biases and to answer the defined clinical question. The choice of the proteomics method and establishing the method performance are of critical importance. Taking appropriate steps to monitor inter-day instrument performance and demonstrating instrument reliability through proper quality control (QC) sample injections is key, as shifts in the liquid chromatographic or mass spectrometric performance can introduce severe interference and add to the experimental variability [159]. Several QC retention time calibrators are currently available to maintain LC MS performance, which can be logged over time in automatic system suitability platforms like AutoQC in Panorama [160]. Given the depth of the analysis and experimental objective of the study, a few to several stages of method validation may be needed to determine the figures of merit (limit of detection, quantification, accuracy, and precision).

To streamline the targeted proteomics measurements, a “fit-for-purpose” approach was used to define three tiers of targeted assays to define the analytical goals of the experiment and the empirical evidence needed to establish that the developed assays perform as intended [161]. The experimental design parameters and assay characteristics (e.g., inclusion of internal standards or reference standards) of each tier were described, with Tier 3 being “exploratory” in nature and a low to moderate degree of analytical validation required; Tier 2 was intended for research grade protein/peptide quantification assays requiring a moderate to high degree of validation; and Tier 1 was described for clinical/diagnostic grade laboratory assays with requirements for a high degree of validation as well as high performance metrics like precision, accuracy, and repeatability. Based on these recommendations and to further ensure transparency and encourage reproducible research, the journal, Molecular Cellular Proteomics, introduced a new set of guidelines for manuscripts describing targeted proteomic measurements [162].

### 5.2. Biomarker Validation

#### 5.2.1. Validation of Discovery Proteomic Data Using Targeted Quantification

DDA-based shotgun proteomics provide an exceptional speed and depth of protein profiling from complex mixtures. This unbiased approach may lead to the identification of novel biomarkers not based on existing hypotheses, and in turn may help generate new hypotheses on disease processes. Explorative proteomic studies using label-free quantification have been used to identify several candidate AD markers. Although the label-free technology requires minimal sample preparation, it suffers from several disadvantages, e.g., this approach is not amenable to multiplexing therefore it results in long LC MS instrument times. This may in turn lead to a lower analytical performance of the instrument over time (retention time shifts, ESI instability leading to inefficient ion transmission). This may be why CSF biomarker studies carried out via label-free methods have lower sample sizes (*n* = 10–20), resulting in lower statistical confidence in the identified markers (A summary of a select discovery proteomic studies for AD biomarker discovery is presented in Table 1). Some of these limitations are overcome by isobaric labeling techniques like TMT, which can greatly eliminate run-to-run differences and where multiplexing is feasible, thereby significantly reducing LC MS analysis time. Although highly reliable, isobaric labeling techniques have some caveats as well, e.g., isotope-labeled reagents can add to the overall cost of the experiment in a large clinical study. Also, additional experimental steps, such as peptide pre-fractionation and verification of labeling completion, may be needed to avoid peptide co-isolation in MS/MS and systematic biases in quantitation. Overall, after comparing the pros and cons of each quantitation strategy for early discovery-based exploratory research, a label-free quantitation approach can analyze a very large number of samples in a single experiment (*n* > 50)—whereas the isobaric labelling techniques may prove cost prohibitive.

**Figure 1:**
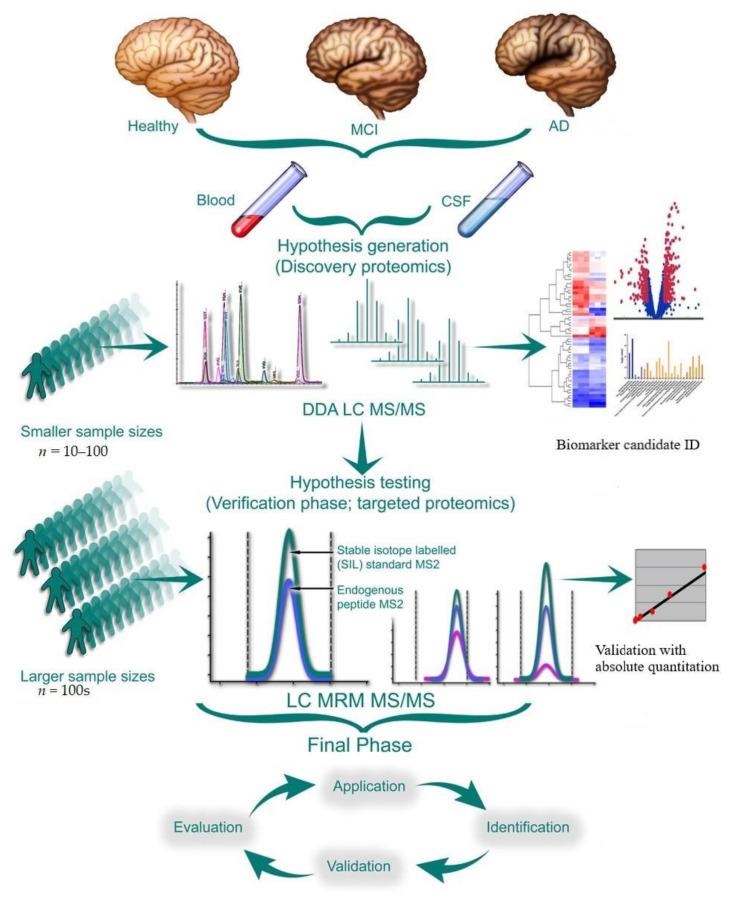
Discovery to validation to clinical implementation of biofluid protein biomarkers in AD: proteomic biomarker discovery pipeline enables an unbiased and untargeted exploration of the biofluid proteome, leading to identification of a list of potential biomarker candidates, which can be validated in larger sample cohorts. After undergoing several rounds of identification, verification, and evaluation, candidate biomarkers can be applied to clinical practice.

Given the inherent limitations of DDA and its stochastic nature, only the highly abundant part of the proteome is reproducibly quantified, and the lower abundant species result in a large number of missing data points. Moreover, the accuracy and consistency of the quantification data further suffers from small sample sizes that do not possess the predictive power of these putative candidates for larger populations. Table 1 presents the summary of a few select proteomic studies that have claimed to identify potential biomarkers of AD in CSF. The quantitative data obtained from such studies undergoes database searching to identify a list of protein identifications and relative quantitation is carried out to identify differential protein expression patterns, which can then be mapped on biological pathways that may be perturbed in the diseased state (Figure 1). These data are further processed downstream. The relative quantitative data obtained from these discovery studies is often unreliable as follow-on validations are not carried out. A recent meta-analysis of 47 unbiased explorative proteomics studies of CSF biomarkers in AD unveiled a panel of 27 proteins and 21 peptides that were highly disrupted in AD in at least three publications [163]. To realize the full potential of these investigations, robust targeted quantitative methods are needed to validate these findings in multiple large independent populations across different stages of the disease.

**Table 1:** Synopsis of published discovery proteomic studies for assay development and biomarker discovery in AD.

Discovery Proteomics Studies			
Sample	LC MS Technique	Summary	Ref.
Plasma AD (*n* = 17) MCI (*n* = 12) Control (*n* = 11)	IP-MS coupled to MALDI-TOF	Immuno-Affinity purification (IP) MS method developed to measure Aβs; Aβ1–40 and Aβ1–42) and Aβ approximate peptides. APP/Aβ (−3–40)/Aβ1–42 ratio was increased in amyloid PET-positive AD patients and was proposed as biomarker to surrogate cerebral amyloid deposition.	[164]
CSF AD (*n* = 100) MCI (*n* = 40) Control (*n* = 80)	Label free LC MS	Anti-neurogranin antibodies were developed and used to show a marked increased level of neurogranin in AD dementia as well as MCI.	[165]
CSF AD patients (*n* = 14) Control (*n* = 14)	IP-PRM-MS	Significantly higher levels of CSF lysosomal protein LAMP2 were reported in AD patients when compared to controls	[166]
CSF Familial AD mutation carries (PSEN1 and APP, *n* = 14) Non-carriers (*n* = 5)	Label free LC MS	Comparative analysis identified 56 significantly differentially-expressed proteins between groups. Fourteen of these aligned with the previous findings. Novel proteins reported include calsyntenin-3, α-amino-3-hydroxy-5-methyl-4-isoxazolepropionic acid receptor, CD99 antigen, di-N-acetyl-chitobiase, and secreted phosphoprotein-1. Protein expression changes in symptomatic and asymptomatic mutation carriers overlapped with those seen in late-onset AD.	[167]
CSF AD patients (*n* = 8) Controls (*n* = 8)	TMT labeling coupled to LC MS	The integrated proteomic and endopeptidomic approach simultaneously analyzed the abundances of 437 endogenous peptides and 374 proteins. The proteins that differed between groups include mesothelin, Ig alpha-1 chain C region, neurexin-1-beta, N-acetyllactosaminide beta-1,3-N-acetylglucosaminyltransferase, neurosecretory protein VGF, isoform 3 of neurotrimin, metalloproteinase inhibitor 2, and UPF0454 protein C12orf49.	[168]
CSF and cultured cells AD patients (*n* = 3) Control (*n* = 3)	CSF and cells combined in the same TMT multiplexed workflow	The optimized TMTcalibrator workflow allowed identification of lowly abundant peptides. Of the 77 proteins identified, 41 that are regulated in AD hadn’t been previously reported.	[169]
CSF AD patients (*n* = 20) Control (*n* = 20)	Endopeptidomic approach with stepwise protein and peptide precipitation followed by MALDI TOF MS and nLC MS	Peptides from VGF nerve growth factor-inducible precursor and α-2-HS-glycoprotein were downregulated in AD and a peptide from complement C4 factor and an O-glycosylated peptide from α-2-HS glycoprotein were found to be elevated	[170]
CSF Healthy volunteers (*n* = 50)	Endopeptidomic approach with TMT labeling coupled to LC MS	Changes in CSF peptidome were measured longitudinally following administration of a γ-secretase inhibitor. Many peptides showed dose-dependent changes in expression, including one derived from APP and one from amyloid precursor-like protein-1, which are known γ-secretase substrates.	[171]
CSF Pooled aliquots (*n* = 14)	Label free LC MS	Quantitative label-free proteomic technique coupled to multi-affinity fractionation was used to assess technical variability as well as inter-subject variation. The technique was also evaluated for its ability to distinguish samples based on the dried biomarker criteria	[172]
CSF Dementia patients (*n* = 159) Controls (*n* = 17)	CE MS to identify differential peptide pattern for early differential diagnosis of various dementias	Using CSF measurements of A β 42, tau, and phospho-tau, the AD pattern was diagnosed with a sensitivity of 87% and a specificity of 83%. Potential synaptic biomarkers identified: Apo-J, chromogranin A, phospholemman, synaptic protein-like proSAAS and neuronal secretory protein VGF	[173]
CSF AD patients (*n* = 4) Controls (*n* = 22)	Label free LC MS	Aβ42 to Aβ40 ratio was estimated in PSEN1 mutant AD using surrogate amyloid precursor-like protein-1-derived Aβ-like peptide (APL1β), including APL1β28. Relatively high ratio of CSF Aβ42 surrogate in PSEN1 mutant AD without an increase of Aβ42 secretion in the brain.	[174]
CSF (*n* = 2)	Label free LC MS for extracellular vesicles (EV) characterization	Exosomal markers identified were alixand syntenin-1, heat shock proteins and tetraspanins and several brain -derived proteins. Known biomarkers of neurodegeneration were also identified in the EV fractions., e.g., amyloid precursor protein, the prion protein, and DJ-1	[175]
CSF Postmortem CSF (*n* = 4) Antemortem CSF (*n* = 4)	TMT 6-plex coupled to LC MS	Discovery analyses found 78 identified proteins to be significantly upregulated in post-mortem CSF samples when compared to antemortem. Previously identified brain damage biomarkers were identified like glial fibrillary acidic protein (GFAP), protein S100B, and protein DJ-1 (PARK7)	[176]
Plasma Non-demented controls (ND, *n* = 36) Non demented subjects with AD family history (ND-FH, *n* = 44) AD (*n* = 40)	Label free LC MS	Aβ-binding proteins circulating in the plasma were isolated and identified by LC MS. Many apolipoproteins were identified, i.e., apoA-I, apoB-100, apoC-III, and apoE. ApoA-I was reduced in AD and was proposed as an AD biomarker. ApoC-III was reduced in both ND-FH and AD and was proposed as a predictive marker for AD	[177]
Plasma Cohort 1 AD (*n* = 24) MCI (*n* = 261) Control (*n* = 411) Cohort 2 MCI (*n* = 180) Control (*n* = 153)	iTRAQ coupled to LC MS	AD-relevant biological pathways enriched in MCI included complement system, the coagulation cascade, lipid metabolism, and metal and vitamin D and E transport. Significant downregulation of potential markers fibronectin and C1 inhibitor was seen in the MCI cohorts.	[178]
Plasma AD (*n* = 15) MCI (*n* = 15) Control (*n* = 15) Validation cohort AD (*n* = 60) Control (*n* = 35)	Isobaric labeling coupled to LC MS	Plasma levels of gelsolin were found to be decreased in AD subjects when compared to controls. This finding was validated via western blotting in the bigger validation cohort. However, additional validation from three different regions of the brain failed to replicate this finding.	[179]
Plasma AD (*n* = 15) Control (*n* = 15)	iTRAQ coupled to LC MS	Differential expression of zinc-alpha-2-glycoprotein (AZGP1), fibulin-1 (FBLN1), platelet basic protein (PPBP), thrombospondin-1 (THBS1), S100 calcium-binding protein A8 (S100A8), and S100 calcium-binding protein A9 (S100A9) seen in the AD patients when compared to controls.	[180]
Plasma Stable MCI (*n* = 58) Progressive MCI (*n* = 34) Control (*n* = 23) AD (*n* = 31)	Label free LC MS	Both inflammation mediating proteins and pro-inflammatory IgG Fc glycoforms were significantly upregulated in AD subjects.	[181]
CSF Delirium (*n* = 17) AD (*n* = 17) Control (*n* = 8)	iTRAQ coupled to LC MS	Discovery analyses of patients with delirium, a risk factor for development of dementia and patients with mild AD identified several interesting protein families, including apolipoproteins, secretogranins, chromogranins, clotting factors, serine protease inhibitors, and acute-phase response elements.	[182]

The sample size (*n*) for each condition is mentioned. The results from discovery studies mentioned were not validated further or were verified using other biochemical techniques, such as western blotting or ELISA.

#### 5.2.2. Higher Throughput Quantitative Assays for Use in Validation Study Cohorts

The validation phase of the biomarker discovery projects aims to overcome some of these limitations of DDA proteomics by allowing for the accurate and consistent quantification of the candidate molecules across large sample cohorts. Follow-up studies are required to validate putative biomarker candidates identified in the discovery datasets. These measures typically use orthogonally-targeted data acquisition schemes such as SRM/MRM or parallel reaction monitoring (PRM), or data independent acquisition (DIA) methods to verify both analyte identification and changes in expression levels with improved quantification [183].

SRM-based targeted proteomics acquisition does not involve full MS2 scans, but rather requires the selection of pre-determined precursor and product ion pairs, known as transitions. The predefined list of transitions for a targeted peptide/protein provides reproducible qualitative and quantitative data for hypothesis testing and biomarker validation studies [184]. Although SRM/MRM analyses provide a high degree of quantitation confidence, they do come at a higher cost of front-end assay development [185]. Standard instruments for SRM/MRM acquisitions include triple quadrupole instruments and quadrupole linear ion traps (QTRAP) operated as triple quadrupoles. PRM provides a compelling alternative to SRM/MRM that provides the same degree of quantitative specificity but a lower burden of method development [186,187]. A PRM experiment involves a full MS2 scan on an orbitrap instrument and lets users pick the optimal fragment ions post-acquisition.

The explorative CSF biomarker studies have benefited greatly from the development of robust and highly-sensitive targeted MRM/PRM methods as verification assays (Figure 1, Table 2). These assays can be developed relatively quickly when compared to immunoassays and remain unbiased from matrix effects and antibody cross-specificity. MRM/PRM methods offer high analyte multiplexing capabilities, although they can be time-consuming when several stages of validation need to be undertaken.

### 5.3. Multisite Variability Assessment: Quantitative Proteomic Data Reporting, Sharing, and the Need for Standardization

In the last few years, we have seen a concerted effort towards establishing accepted protocols, guidelines, and criteria for the testing, reporting, and qualification of mass spectrometry-based proteomic biomarkers. Multi-site replication studies are needed for the assessment of intra-lab and inter-lab variability by utilizing the same or alternative proteomic technique and data processing platforms. Research transparency is encouraged through data sharing to scientists, regulators, and clinicians through online repositories, such as ProteomeXchange (Pride [199,200]) and Panorama Public [201]. LC MS AD biomarker discovery research can borrow conventional wisdom from the more mature oncology initiatives, such as Clinical Proteomic Tumor Analysis Consortium (CPTAC). CPTAC is a web-based assay portal that aims at advancing the development and dissemination of thoroughly validated mass spectrometry-based targeted proteomic assays. CPTAC also actively supports proteogenomic translational research to integrate, visualize, and analyze cancer biology across multiple ‘omics’ data dimensions like genomics, transcriptomics, and proteomics [202]. The CPTAC data portal provides a central database for the sharing and re-use of data across the research community to advance cancer biomarker discovery and clinical translation [203]. Moreover, CPTAC has provided a validation guidance document to help researchers carry out MRM assay characterization, thus promoting high standards for assay inclusion. The validation document outlines five experiments to evaluate assay parameters like linearity, intra-assay precision, upper and lower limit of quantification, repeatability, selectivity, and internal standard stability [204].

## 6. Case Study—Longitudinal Proteomic Changes in CSF from ADNI: Towards Better Defining the Trajectory of Early Alzheimer’s Disease

The Alzheimer’s Disease Neuroimaging Initiative (ADNI), set up in 2004, is an ongoing multicenter project that has come a long way to identify changes in brain structure and function, as well as cognition-related changes [205]. One of the major objectives of ADNI has been to validate MRI, PET, and CSF/blood biomarkers as outcome predictors to be used in AD clinical trials. ADNI has made a profound impact in the field through the development and implementation of standardized protocols across multiple sites and improved clinical trial efficiency by the identification of sensitive outcome measures for patient stratification [206,207]. The overarching goal of ADNI is to detect AD at the earliest possible stage and implement ways to stage AD progression using robust biomarkers [208]. Another defining characteristic of ADNI is its innovative data-access policy and the commitment to allow embargo-free data sharing to stimulate collaborations and further scientific investigations to answer unresolved questions about competing hypotheses regarding disease pathophysiology [209]. The Laboratory of Neuroimaging (LONI) at the University of Southern California hosts all the archives of the data generated via ADNI studies. Due to the open data sharing approach, presently over 1000 publications have utilized ADNI data in AD research as well as in fields outside of AD [186]. ADNI data has also been used by big data projects and a number of consortia, such as the Enhancing Neuro Imaging Genetics through Meta-Analysis (ENIGMA) consortium [210], as well as Dialogue on Reverse Engineering Assessment and Methods (DREAM) Alzheimer’s disease Big Data Challenge #1 [211].

ADNI samples have been utilized in a one-of-a-kind, multi-phased effort, using targeted multiplexed mass spectrometry-based assays to identify diagnostic and predictive CSF-based biomarkers in AD. This project presented the analysis of many candidate peptide markers from a well-characterized AD patient sample cohort. Phase 1 of this study was conducted as a feasibility analysis to assess MRM assay characteristics, e.g., the reproducibility of sample processing, analytic variability, and the ability to detect a variety of analytes of interest. The CSF multiplex MRM panel was built with proteins and peptides curated from a variety of published and unpublished AD biomarker studies in CSF, brain and cell lines, and previous results from the Rules Based Medicine (RBM) multiplex ADNI CSF immunoassays. Phase 1 of the study was completed using 25 human CSF samples that included 5 technical replicates, which underwent abundant protein depletion followed by tryptic digestion and were detected by LC MRM MS. Of the 510 peptides/267 proteins queried, 198 peptides/121 proteins were detectable in the CSF, measuring 2 transitions per peptide. After the successful completion of the pilot study, phase 2 was initiated. The final MRM panel was built by supplementing the detectable peptides from the pilot study with many other peptides, particularly from proteins of interest that were either inflammatory markers or those of interest in the RBM assay. The final MRM assay consisted of 567 peptides representing 222 proteins. A system suitability test of the LC MRM MS system was carried out using synthetic standard peptide solutions to assess instrument reproducibility as well as sensitivity. Several filtering criteria were employed to refine the final MRM transitions using parameters such as co-elution with the standard, retention time stability over runs, reproducibility of the intensity ratios, and setting a detection threshold. The refined MRM assays were applied to 306 ADNI-1 CSF samples (85 healthy controls, 66 AD patients, and 134 MCI patients), which included 16 blinded technical replicates. A variety of statistical approaches, such as univariate association/prediction analyses as well as multivariate exploratory/supervised analyses, were employed to assess whether the analytes were associated with clinical pathology like MCI or AD vs. healthy controls or associated with progression vs. non-progression from MCI to AD. The results included several peptides with potential “diagnostic” utility, mainly from Hemoglobin A (HBA), Hemoglobin B (HBB), and superoxide Dismutase (SODE), as well as peptides with “predictive” utility from neuronal pentraxin-2 (NPTX2), neurosecretory protein VGF (VGF), and secretogranin-2 (SCG2) [212]. This study utilized extensive quality control and data normalization measures and applied a level of rigor that has not been previously demonstrated in proteomic biomarker research.

The next phase of this effort focuses on within-subject alterations over time in patients with MCI, AD, and healthy controls. This longitudinal sample set has at least three CSF samples from each patient drawn over a period of three years or more. The aim of this project was to evaluate the ability of a panel of peptides to distinguish the disease states and show changes in a longitudinal manner. Proteins and peptides were selected based on their previous detection in CSF, AD relevance, and previous results from RBM, as well as earlier MRM assays. A total of 5 primary (Fatty Acid Binding Protein-3 (FABP3), SCG2, VGF, NPXT2, and Chromogranin A (CHGA)) targets were chosen for absolute quantification and 121 targets were chosen for relative quantification. The final MRM assay consisted of 278 peptides representing 126 proteins. The refined MRM assays were applied to 750 unique CSF samples that were comprised of 730 longitudinal samples from ADNI-1, ADNI-2, and ADNI-GO, as well as 20 blinded replicates. The samples were processed in a randomized and blinded manner. Due to the presence of endogenous proteins in CSF, the standard curve for absolute quantitation was built using recombinant proteins (FABPH, SCG2, VGF, NPXT2, and CHGA) in BSA buffer. Quality control samples were prepared using a CSF pool of 300 individual CSF samples supplied by ADNI. The blinded phase of the project has been completed (unpublished data) and further analysis using linear mixed-effects modeling is underway as ADNI subject IDs are un-blinded. The primary objective of this study is to retrospectively investigate analytes that exhibit intra-individual trajectories corresponding to various stages of disease, including cognitively normal, MCI, and AD dementia. The data indicated that neuronal pentraxin 2 (NPTX2) could be a potential disease progression marker in AD. NPTX2 exhibited a robust association with baseline clinical diagnosis of MCI for the intra-subject evaluations. Moreover, degradation of NPTX2 showed a positive correlation with cognitive decline. These findings suggested that NPTX2 could be a potential prognostic biomarker of accelerated cognitive decline in a subset of AD patients [213].

These ADNI studies demonstrate the potential for the discovery of novel biomarkers that can be used as endpoints in clinical trials of early AD, can help monitor treatment effects, enable more efficient trial design, and further help understand the therapeutic mechanism of action.

## 7. The Promise of Fluid Biomarkers of CNS-Related Diseases

The paucity of early detection and disease progression biomarkers seems to present a major obstacle to AD drug development. Current diagnostic measures based on neuroimaging-based techniques, such as structural and functional Magnetic Resonance Imaging (MRI) and Positron Emission Tomography (PET), are expensive and require advanced on-site technologies and expertise. There is an urgent need to identify new markers from biofluids such as CSF and blood, which can serve as diagnostic markers of the disease and clinical endpoints for new drugs being tested in clinical trials. These biofluid biomarkers can be attained through less invasive means and measures are amenable to routine clinical laboratory workflows. Traditional biochemical techniques generally focus on only a few key genes/proteins to investigate molecular processes, the resulting data often provides an insufficient and somewhat incomplete understanding of the complex intricacies of AD pathology. Mass spectrometry-based multiplexed targeted proteomic assays can be used as an efficient strategy to analyze many biofluid candidate markers with diagnostic and prognostic value for AD drug development. As demonstrated by the large-scale ADNI studies, the promise of novel CSF AD biomarkers can be realized by carrying out large-scale, longitudinal, well-characterized, robust studies with good statistical power.

## Figures and Tables

**Table 2:** Synopsis of relevant targeted proteomic studies for biomarker validation in AD.

Discovery Proteomics Studies			
Sample	LC MS Technique	Summary	Ref.
CSF AD dementia (*n* = 8), MCI (*n* = 11), controls (*n* = 19)	TMT coupled to IP-MS	Robust assay developed for parallel relative quantification of 27 Aβ peptides in CSF. Although no statistical difference was seen between diseased and control groups.	[188]
CSF and blood AD patients (*n* = 39) Control patients (*n* = 38)	SRM: Absolute quant with heavy isotope standards	ApoE proteoforms quantified using stable isotope dilution. Total ApoE in CSF or blood doesn’t distinguish AD from non-AD subjects. ApoE e4 carriers have lower blood ApoE irrespective of clinical diagnosis.	[189]
Plasma Case-control (*n* = 669)	SRM-MS	Total ApoE and ApoE e4 proteoform quantified. ApoE e4 specific peptide contained a single methionine, which was chemically oxidized after tryptic digestion, completeness of oxidation was thoroughly evaluated. Chemical oxidation allowed unbiased monitoring of ApoE e4 unique proteotypic peptide. Neither total ApoE and ApoE e4 levels nor ApoE/APOE e4 ratio consistent with AD diagnosis	[190]
Serum DLB patients (*n* = 47) AD patients (*n* = 97)	SpotLight Melon Gel kit enriches polyclonal IgGs.	De-novo sequencing identifies peptides from variable regions of IgGs and uncovers “hidden proteome”. SpotLight peptide quantification generated a predictive model with 95% accuracy to distinguish AD and dementia with Lewy bodies	[191]
CSF (Sample size not listed)	IP-MS with heavy isotope internal standards coupled to MALDI-TOF. Confirmation carried out on LIT-FT ICR	Affinity purification MS method optimized for Aβ using Aβ specific crosslinked antibodies. Two novel Ab peptides identified: Aβ2-17 and Aβ3-17 (probable cleavage products of neprilysin and ECE) The developed assay facilitated target engagement clinical studies	[192,193,194]
CSF (three separate cohorts) Cohort 1: AD subjects (*n* = 9), prodromal AD (*n* = 7), non-demented controls (*n* = 9) Cohort 2: AD (*n* = 10), non-demented controls (*n* = 6) Cohort 3: AD (*n* = 17), non-demented controls (*n* = 17) Brain tissue Autopsy confirmed AD patients (*n* = 15) Age-matched controls (*n* = 15)	IP-SRM-MS with heavy isotope internal standards	Affinity purification MS method developed to measure levels of the presynaptic protein synaptosomal-associated protein 25 (SNAP-25) in CSF. SNAP-25 levels were significantly higher in prodormal AD and AD when compared to controls. CSF SNAP-25 differentiated AD from controls and was proposed as novel biomarker for synapse degeneration.	[195]
CSF (2 cohorts) Cohort 1: CSF AD dementia (*n* = 15), MCI (*n* = 5), controls (*n* = 17) Cohort 2: CSF AD (*n* = 24), MCI (*n* = 18), controls (*n* = 36)	IP-PRM-MS with heavy isotope internal standards	Affinity purification MS method developed to measure levels of the presynaptic vesicle protein synaptotagmin-1 in CSF Synaptotagmin-1 levels were significantly higher in MCI AD and AD dementia when compared to controls. CSF synaptotagmin-1 was proposed as a biomarker of synaptic dysfunction and degeneration in AD	[196]
CSF and plasma AD patients (*n* = 43) Control (*n* = 43)	SRM MS with heavy isotope internal standards	Previously developed ApoE quantification assay was used to measure ApoE proteoforms ApoE2, ApoE3 and ApoE4. No distinction was found between AD patients aid controls.	[197]
CSF AD patients (*n* = 37) Control (*n* = 22) Validation cohort AD patients (*n* = 24) Control (*n* = 16)	SRM with heavy isotope internal standards	Significantly higher concentration of soluble triggering receptor expressed on myeloid cells 2 (sTREM2) was found in AD patients when compared to controls. This finding was replicated in the validation sample set. sTREM2 was found to correlate with markers of neurodegeneration and glial activation.	[198]

The sample size (*n*) for each condition is mentioned.
